# The Tumorigenic Effect of lncRNA AFAP1‐AS1 is Mediated by Translated Peptide ATMLP Under the Control of m^6^A Methylation

**DOI:** 10.1002/advs.202300314

**Published:** 2023-03-04

**Authors:** Hailong Pei, Yingchu Dai, Yongduo Yu, Jiaxin Tang, Zhifei Cao, Yongsheng Zhang, Bingyan Li, Jing Nie, Tom K. Hei, Guangming Zhou

**Affiliations:** ^1^ State Key Laboratory of Radiation Medicine and Protection School of Radiation Medicine and Protection Suzhou Medical College of Soochow University Jiangsu Suzhou 215123 P. R. China; ^2^ Department of Pathology The Second Affiliated Hospital of Soochow University Jiangsu Suzhou 215004 P. R. China; ^3^ Center for Radiological Research College of Physician and Surgeons Columbia University New York NY 10032 USA

**Keywords:** autolysosomes, ionizing radiation, mitophagy, N6‐methyladenosine, tumorigenesis

## Abstract

Long noncoding RNAs (lncRNAs) in eukaryotic transcripts have long been believed to regulate various aspects of cellular processes, including carcinogenesis. Herein, it is found that lncRNA *AFAP1‐AS1* encodes a conserved 90‐amino acid peptide located on mitochondria, named lncRNA *AFAP1‐AS1* translated mitochondrial‐localized peptide (ATMLP), and it is not the lncRNA but the peptide that promotes the malignancy of nonsmall cell lung cancer (NSCLC). As the tumor progresses, the serum level of ATMLP increases. NSCLC patients with high levels of ATMLP display poorer prognosis. Translation of ATMLP is controlled by m^6^A methylation at the 1313 adenine locus of *AFAP1‐AS1*. Mechanistically, ATMLP binds to the 4‐nitrophenylphosphatase domain and non‐neuronal SNAP25‐like protein homolog 1 (NIPSNAP1) and inhibits its transport from the inner to the outer mitochondrial membrane, which antagonizes the NIPSNAP1‐mediated regulation of cell autolysosome formation. The findings uncover a complex regulatory mechanism of NSCLC malignancy orchestrated by a peptide encoded by a lncRNA. A comprehensive judgment of the application prospects of ATMLP as an early diagnostic biomarker for NSCLC is also made.

## Graphical abstract

Noncoding RNA is widely considered without coding capability, and the traditional gene annotation process filters out proteins that are less than 100 amino acids in length. This makes us lose many proteins encoded by micro‐open reading frames in long noncoding RNA. These findings break the traditional understanding and successfully identify the *AFAP1‐AS1* translated mitochondrial‐localized peptide, which can be used as early diagnostic biomarker for NSCLC.

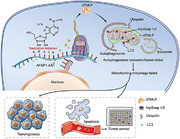

## 1 Introduction

The mammalian genome produces tens of thousands of noncoding transcripts during the transcription process. Approximately 98% of RNAs in the human transcriptome are noncoding. Traditionally, noncoding RNAs (ncRNAs) are transcribed from the genome but not translated into proteins. Emerging evidence suggests that long noncoding RNAs (lncRNAs) play a number of cellular functions, including the regulation of transcriptional activation,^[^ ^1^ ^]^ chromosome inactivation,^[^ ^2^ ^]^ heterochromatin formation,^[^ ^3^ ^]^ and the maintenance of telomeres.^[^ ^4^ ^]^ Alterations in some lncRNAs have been shown to regulate important cancer hallmarks, including metabolism,^[^ ^5^ ^]^ tumorigenesis^[^ ^6^ ^]^ and drug/radiation resistance.^[^ ^7^ ^]^ NcRNAs display tissue‐specific expression patterns and are potential biomarkers. Thus, they could serve as clinical diagnostic and prognostic indicators.^[^ ^8^ ^]^

Noncoding RNA (ncRNA) is widely described as a class of RNA molecules transcribed from genomic DNA without coding capability.^[^ ^9^ ^]^ Additionally, the traditional gene annotation process filters out proteins that are <100 amino acids in length and treats them as “noise” or false‐positives.^[^ ^10^ ^]^ Hence, we consistently ignored the group of small “noise proteins” encoded by “genomic noise.” Recent advances in bioinformatics and biochemical methodologies have revealed that ncRNAs that were previously considered noncoding may, in fact, encode small biologically active peptides.^[^ ^9^, ^11^, ^12^, ^13^ ^]^ These coding products play important roles in the pathogenesis of many diseases, such as regulating intracellular ion transport,^[^ ^14^ ^]^ immunity or inflammation,^[^ ^15^, ^16^ ^]^ muscle regeneration,^[^ ^17^ ^]^ lipid metabolism,^[^ ^18^ ^]^ and tumorigenesis.^[^ ^19^, ^20^ ^]^

There is evidence that the small open reading frame (ORF) can be found within a transcript annotated as a long noncoding RNA (lncRNA).^[^ ^21^, ^22^ ^]^ To identify potential peptides, we searched presumed ncRNA transcripts for hypothetical ORFs using PhyloCSF and ORF Finder (NCBI), which focuses on codon substitution frequencies.^[^ ^23^ ^]^ From these transcripts, we discovered a previously unrecognized ORF of 90 amino acids, which we named lncRNA AFAP1‐AS1 translated mitochondrial‐localized peptide (ATMLP). The lncRNA AFAP1‐AS1 RNA transcript is annotated as NR_026892.1 in the NCBI Reference Sequence and ENSG00000272620 in Ensembl. With only 90 amino acids, ATMLP is currently a new peptide known to be encoded by a lncRNA located in mitochondria. Here, we show that ATMLP increases the malignant transformation of epithelial cells and tumorigenesis by promoting incomplete cell mitophagy.

## 2 Results

### 2.1 AFAP1‐AS1 Encodes a Micropeptide

**Figure 1:**
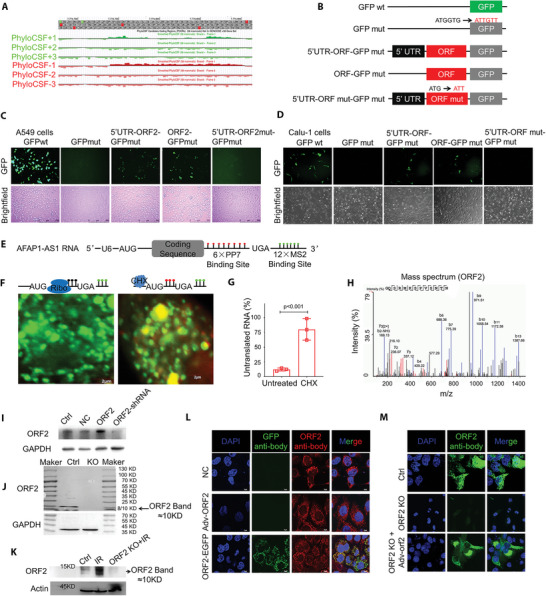
AFAP1‐AS1 encodes a novel peptide. A) PhyloCSF score plot for the AFAP1‐AS1 ORF2 peptide (also called ORF2) locus as seen in the UCSC genome browser using the PhyloCSF track hub. B) Diagram of the GFP fusion constructs used for transfection. The start codon ATGGTG of the GFP (GFPwt) gene is mutated to ATTGTT (GFPmut). The start codon ATG of the AFAP1‐AS1 ORF2 is mutated to ATT. C,D) The indicated constructs were transfected into A549 C) and Calu‐1 D) cells for 24 h, and GFP fluorescence was observed as a surrogate marker for protein expression after 24 h. E) Schematic of the TRICK reporter transcript. 6×PP7 stem‐loops inserted in‐frame with the C‐terminus of a protein‐coding sequence and 12×MS2 stem‐loops in the 3′UTR. F) Cytoplasmic region of untreated and cycloheximide (100 µg mL^−1^)‐treated Calu‐1 cells. Scale bar, 2 µm. G) Percentage of untranslated TRICK mRNAs in Calu‐1 cells. H) Mass spectrum of the AFAP1‐AS1 ORF2 peptide sequence. I,J) Verification of the AFAP1‐AS1 ORF2 peptide antibody. NC: Negative control, OE: Overexpressing AFAP1‐AS1 ORF2 peptide with adenoviruses. KO: AFAP1‐AS1 ORF2 knockout. K) Radiation‐induced AFAP1‐AS1 ORF2 peptide expression. L,M) Immunofluorescence was used to verify the translation of the cytoplasmic peptide of the AFAP1‐AS1 ORF2 peptide antibody. The AFAP1‐AS1 ORF2 peptide‐EGFP fusion protein and overexpressing adenovirus were used as positive controls.

Ionizing radiation induces a number of cellular and genetic changes, including the DNA damage response, cell death and genomic alterations, including changes in both coding and noncoding RNAs among both normal and cancer cells. To define the potential connection of lncRNAs in radiation‐induced tumorigenesis, we checked the differentially expressed lncRNAs in irradiated lung epithelial cells. After exposure to a 2 Gy dose of X‐rays, 365 significantly differentially expressed (fold change >2, *p* < 0.05) lncRNAs were identified, among which 71 were distinctively downregulated, while 294 were upregulated (Figure S1A, Supporting Information). Among the lncRNAs identified (after X‐ray treatment), *AFAP1‐AS1* (XR_026892.1 in NCBI or ENSG00000272620 in Ensembl) was closely related to radiation‐induced cell biological effects. It has been reported that *AFAP1‐AS1* is an oncogene.^[^ ^21^ ^]^ Bioinformatics analysis predicted that *AFAP1‐AS1* is highly expressed in NSCLC and is often associated with poor prognosis (Figure S1B,C, Supporting Information). The human *AFAP1‐AS1* gene consists of two exons spanning 6.81 kilobases (kb), while all the predicted ORFs are located in exon 2. We searched presumed ncRNA transcripts for hypothetical ORFs using PhyloCSF, a method that uses codon substitution frequencies to identify potential peptides (**Figure** **1**A and Figure S2A,B, Supporting Information). Although annotated as a lncRNA, our analysis of ribosome‐profiling datasets incorporated with GWIPS‐viz revealed ribosome occupancy in the lncRNA *AFAP1‐AS1* transcript (Figure S2C, Supporting Information), hinting that lncRNA *AFAP1‐AS1* might be translated. Using the open reading frame (ORF) finder, we identified seven potential ORFs (Figure S2D, Supporting Information). To test the coding potential of *AFAP1‐AS1*, we generated several constructs in which a GFP‐mutation (the first two codons in GFP, ATGGTG, are mutated to ATTGTT) sequence was fused to the C‐terminus of these ORFs in the *AFAP1‐AS1* transcript (Figure 1B and Figure S2E, Supporting Information). From these transcripts, we discovered a previously unrecognized ORF of 90 amino acids, which we called the *AFAP1‐AS1* ORF2 peptide (Figure S2F, Supporting Information, AFAP1‐AS1 peptide or ORF2 in Figures). Substantial expression of the AFAP1‐AS1‐ORF2‐GFP fusion protein was also observed in A549 cells (Figure 1C) and Calu‐1 cells (Figure 1D) transfected with AFAP1‐AS1 ORF2‐GFPmut and 5′‐UTR‐ORF2 GFPmut. However, mutation of the AFAP1‐AS1 ORF2 start codon from ATG to ATT (5′‐UTR ORFmut‐GFPmut) abolished the expression of the AFAP1‐AS1 ORF2‐GFP fusion protein (Figure 1C,D). Because the ribosome or its associated factors must displace endogenous RNA‐binding proteins during the first round of translation, we constructed an RNA biosensor whose fluorescent signal would depend on this process. The stem‒loop sequence of orthogonal bacteriophage PP7 was fused with ORF2 of *AFAP1‐AS1*, and an MS2 stem‒loop sequence was tagged at the 3′UTR. Simultaneous expression of the PP7 coat protein fused to a nuclear localization sequence (NLS) and red fluorescent protein (NLS‐PCP‐RFP) and the MS2 coat protein fused to an NLS and green fluorescent protein (NLS‐MCP‐GFP) resulted in nuclear transcripts labelled with both fluorescent proteins (Figure 1E). The results showed that almost all of the *AFAP1‐AS1* RNA appeared as green particles in the cytoplasm, indicating that only NLS‐MCP‐GFP was bound (Figure 1F). Quantification revealed that 87.65% of reporter *AFAP1‐AS1* RNA had been translated at least once (Figure 1F,G). To confirm that loss of NLS‐PCP‐RFP from cytoplasmic transcripts was translation dependent, we added cycloheximide, which inhibits elongation, for 30 min before induction of reporter *AFAP1‐AS1* RNA expression and found an increase in the number of untranslated RNAs in the cytoplasm (Figure 1F,G). The results of the mass spectrum also showed that partial sequences (GQSRMKSPVSNTN) of the AFAP1‐AS1 ORF2 peptide could be identified (Figure 1H). To detect the endogenously produced peptide encoded by the AFAP1‐AS1 ORF2 peptide in cells, we designed and produced an AFAP1‐AS1 ORF2 peptide antibody. In the following western blotting verification studies, we used wild‐type and AFAP1‐AS1 ORF2 peptide overexpressing, knockdown or knock‐out cells to verify the presence of the peptide. The results showed that the AFAP1‐AS1 ORF2 peptide antibody could effectively hybridize both endogenous and overexpressed AFAP1‐AS1 ORF2 peptide (Figure 1I,J). We used 2 Gy X‐rays to treat BEAS‐2B cells and found that radiation could significantly induce AFAP1‐AS1 ORF2 peptide expression, but the expression of AFAP1‐AS1 ORF2 was significantly neutralized after X‐ray exposure in AFAP1‐AS1 ORF2 knockout cells (Figure 1K). We further used the immunofluorescence approach described above to verify the translation of cytoplasmic peptide and obtained the same result (Figure 1L,M and Figure S3, Supporting Information). Together, these results reveal that AFAP1‐AS1 RNA, which is annotated as noncoding, in fact encodes a cryptic peptide.

### 2.2 High Levels of Endogenous AFAP1‐AS1 ORF2 Peptide Correlate with Poor Prognosis for NSCLC Patients

**Figure 2:**
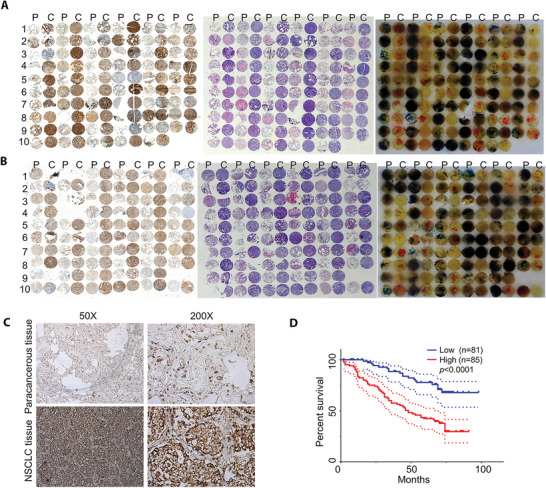
High levels of AFAP1‐AS1 ORF2 peptide are associated with poor prognosis in NSCLC patients. A,B) Representative IHC and HE images of AFAP1‐AS1 ORF2 peptide expression in NSCLC tissues and corresponding paracancerous tissues. Left panel: IHC image. Middle panel: HE staining image. Right panel: hartparaffin images. P: paracancerous tissues. C: NSCLC tissues. C) Enlarged figures of IHC images in NSCLC tissues and corresponding paracancerous tissues. D) Verification of differences in AFAP1‐AS1 ORF2 peptide expression scores between NSCLC tissues and corresponding paracancerous tissues. Kaplan‒Meier survival analysis of NSCLC patients was performed based on AFAP1‐AS1 ORF2 peptide expression ratios of cancer/paracancer tissues (*n* = 166). ORF2 in the figures indicates the AFAP1‐AS1 ORF2 peptide.

To examine the role of the AFAP1‐AS1 ORF2 peptide in NSCLC carcinogenesis, we collected 196 pairs of NSCLC cancer tissues and surrounding paracancerous tissues and performed extensive immunohistochemical (IHC) and HE analyses on tissue microarrays. AFAP1‐AS1 ORF2 peptide levels were found to be significantly upregulated in NSCLC tissues compared with paracancerous tissues (**Figure** **2**A–C and Figure S4, Supporting Information). Furthermore, upregulated AFAP1‐AS1 ORF2 peptide levels were positively associated with more advanced disease in NSCLC (*p* = 0.037). Kaplan‒Meier survival analyses revealed an inverse correlation between overall survival and the expression level of the AFAP1‐AS1 ORF2 peptide (Figure 2D, *p* < 0.0001, log‐rank test). The mean overall survival time for NSCLC patients with high levels of AFAP1‐AS1 ORF2 peptide was only 46.0 months, whereas survival for NSCLC patients with low levels of AFAP1‐AS1 ORF2 peptide was 72.0 months. Therefore, upregulated AFAP1‐AS1 ORF2 peptide levels correlated with poor overall prognosis in NSCLC patients.

### 2.3 *AFAP1‐AS1* m^6^A Methylation Promotes AFAP1‐AS1 ORF2 Peptide Translation

**Figure 3:**
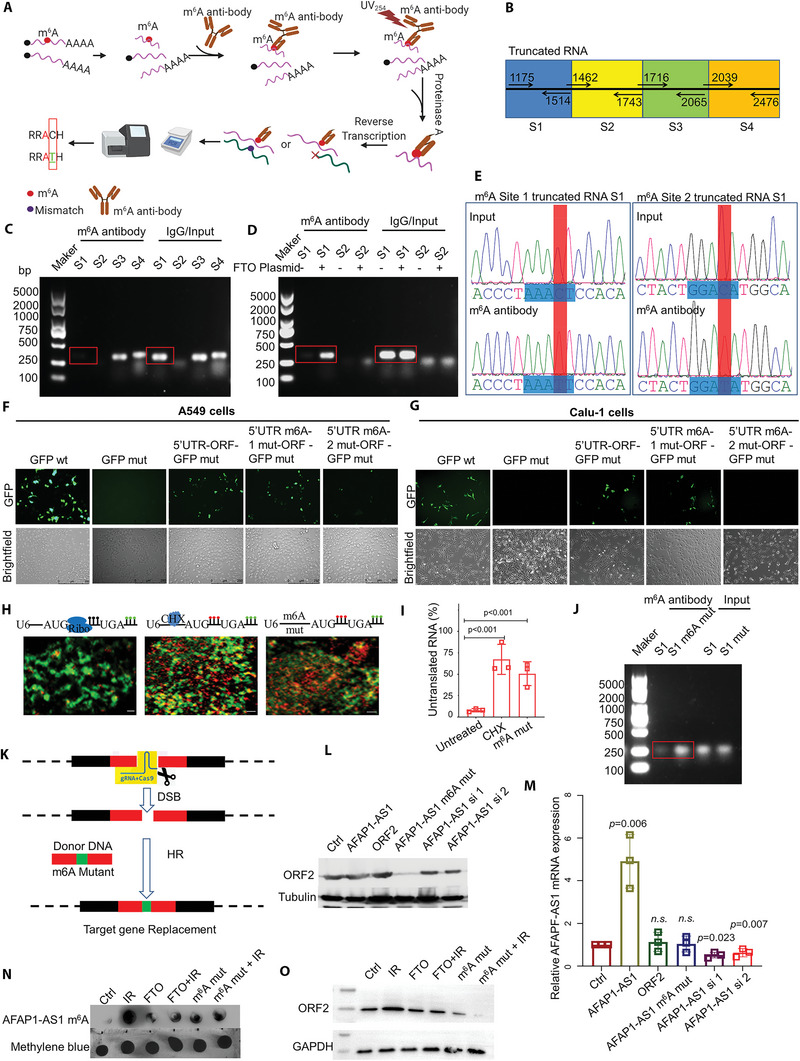
*AFAP1‐AS1* m^6^A methylation promotes AFAP1‐AS1 ORF2 peptide translation. A) The miCLIP protocol. Purified cellular RNA was fragmented and incubated with anti‐m^6^A. After crosslinking with UV light (254 nm), covalently bound antibody‐RNA complexes are recovered by protein A/G affinity purification, PAGE, and nitrocellulose membrane transfer. RNA is then released from the membrane by proteinase K and reverse‐transcribed. Peptide fragments that remain on the RNA lead to nucleotide incorporation errors (indicated as C→T transitions) and cDNA truncations. B) Schematic diagram of fragment design for m^6^A detection in the *AFAP1‐AS1* gene. C,D) PCR product (DNA) analysis by gel electrophoresis. cDNA was obtained in cells with FTO D) or without FTO C). E) Sequence information for the PCR product (DNA). F,G) The indicated constructs were transfected into A549 and Calu‐1 cells for 24 h, and GFP fluorescence in A549 cells F) and Calu‐1 cells G) was measured. H) Cytoplasmic region of untreated, cycloheximide (100 µg mL^−1^)‐treated and m^6^A mutant Calu‐1 cells. Scale bar H), 0.8 µm. I) Percentage of untranslated TRICK mRNAs in Calu‐1 cells. J) PCR product (DNA) analysis by gel electrophoresis. cDNA was obtained from cells with and without the m^6^A site mutation. K) Diagram of the constructs used for genome m^6^A point mutation. L,M) Endogenous AFAP1‐AS1 ORF2 peptide L) or *AFAP1‐AS1* RNA M) was detected by western blotting or PCR in A549 cells. N) M^6^A Dot Blot experiment detecting the m^6^A modification level of AFAP1‐AS1 in BEAS‐2B cells. O) Endogenous AFAP1‐AS1 ORF2 peptide was detected by western blotting in BEAS‐2B cells.

For nonencoded RNA without a 5′‐cap, cap‐independent translation regulated by m^6^A may play the role of the translation initiation complex. Herein, we used a m^6^A antibody generated against UV‐induced m^6^A to crosslink RNA followed by reverse transcription to identify the m^6^A site on *AFAP1‐AS1* using the standard m^6^A single‐nucleotide resolution UV crosslinking and immunoprecipitation (miCLiP) method (**Figure** **3**A). To define sites of m^6^A methylation, we analyzed sliding windows (length, 500 nt; step size, 10 nt) that overlapped with annotated RefSeq transcripts for m^6^A site coverage on lncRNA *AFAP1‐AS1*. We divided the lncRNA *AFAP1‐AS1* sequence into four parts based on the number of m^6^A methylation sites. Finally, the number of miCLIP‐called m^6^A residues in each cluster was determined by PCR amplification (Figure 3B). Sequence S1 rarely accumulated with the m^6^A antibody (Figure 3C). We then eliminated the methylation sites in the genome using the RNA demethylase FTO and found that S1 could now be enriched by m^6^A antibody again (Figure 3D). This confirmed that the S1 sequence possessed potential m^6^A methylation sites. Previous reports demonstrated that antibodies recognizing m^6^A can induce specific C to T transition mutational signatures at m^6^A residues after UV light‐induced antibody‐RNA crosslinking and reverse transcription. As such, we collected the S1 sequence enriched by the m^6^A antibody and, through PCR product sequencing, identified the two potential m^6^A sites of the S1 sequence that included a C to T mutation (Figure 3E). To verify which of the two potential m^6^A sites was the essential site that controls the translation of the lncRNA, we designed adenine mutations at these two potential m^6^A sites. After the 1311–1315 GGACC sequence mutation, enhanced GFP (EGFP) fusion protein expression was significantly reduced in A549 cells (Figure 3F) and Calu‐1 cells (Figure 3G). To further verify whether the lncRNA *AFAP1‐AS1* can be translated, we designed a mutant of 1311–1315 GGACC in *AFAP1‐AS1* RNA. The RNA translation biosensor showed that cycloheximide‐treated cells retained 68.68 ± 18.4% of untranslated AFAP1‐AS1 RNA, and *AFAP1‐AS1* m^6^A mutant RNA retained 49.65±15.65% of untranslated RNA, while in the *AFAP1‐AS1* wild‐type group, only 8.12 ± 4.22% of untranslated RNA remained (Figure 3H,I). This result indicated that m^6^A methylation promotes the translation of lncRNAs. We also found that the 1311–1315 GGACC mutant could not be further enriched by the m^6^A antibody (Figure 3J). To investigate the influence of the AFAP1‐AS1 ORF2 peptide and lncRNA on cancer progression, we constructed an A549 cell line with a genome harboring the 1311–1315 GGACC mutation using the CRISPR/Cas9 method with donor DNA (Figure 3K). Additionally, the AFAP1‐AS1 peptide was transfected into A549 cells. The results showed that the 1311–1315 GGACC m^6^A mutation induced *AFAP1‐AS1 ORF2* knockout but not its mRNA (Figure 3L,M), confirming that the m^6^A site at position 1311–1315 is the core sequence that controls the translation of the AFAP‐AS1 peptide. To clarify the impact of AFAP1‐AS1 m^6^A modification under the pressure of radiation, we detected the content of the m^6^A modification with DotBlot assay in BEAS‐2B cells. The results showed that ionizing radiation could significantly increase AFAP1‐AS1 m^6^A modification. However, after overexpressing FTO in cells, the impact of radiation on the m^6^A content was rapidly decreased. Simultaneously, the m^6^A content also decreased significantly in AFAP1‐AS1 1311–1315 GGACC m^6^A mutant cells. AFAP1‐AS1 ORF2 peptide expression was consistent with the m^6^A content results. As a result, radiation could increase the expression of the AFAP1‐AS1 ORF2 peptide by enhancing AFAP1‐AS1 m^6^A modification.

### 2.4 AFAP1‐AS1 ORF2 Peptide, but not *AFAP1‐AS1* lncRNA, Suppresses Autolysosome Formation, Which Leads to Incomplete Mitophagy in NSCLC Cells

**Figure 4:**
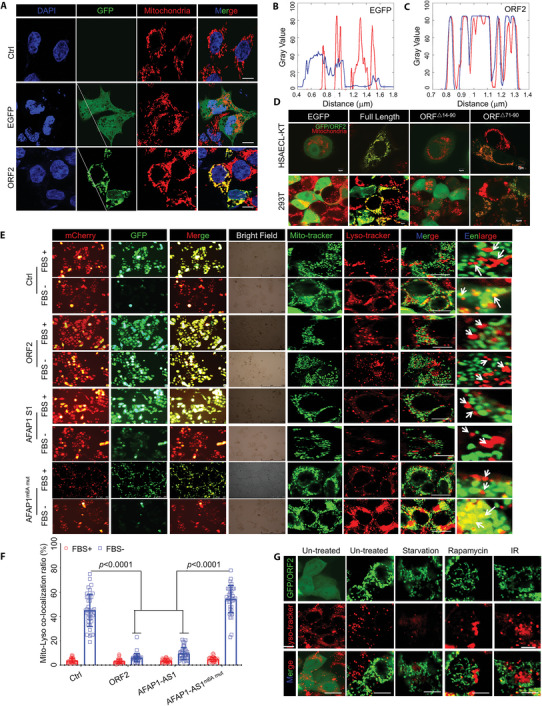
AFAP1‐AS1 ORF2 peptide, but not lncRNA *AFAP1‐AS1*, suppresses NSCLC cell mitophagy. A–C) The indicated AFAP1‐AS1 ORF2 peptide GFP fusion protein was expressed in A549 cells. Mitochondria showed COX‐4 antibody hybridization. The colocalization of the two channels was calculated by ImageJ software. D) Truncated AFAP1‐AS1‐ORF2‐EGFP fusion protein was expressed in HSAEC1‐KT and HEK‐293T cells. E,F) A549 cells were transfected with the indicated LC3B‐mCherry‐EGFP dual‐fluorescence constructs, and the mitophagy rate and autophagolysosome formation rate were measured. The white arrows indicate lysosomes and mitochondria that have or have not been positioned. G) A549 cells were treated with different stress conditions, and the autophagolysosome formation rate was measured. Data are represented as the means ± SEMs. ORF2 in the figures indicates the AFAP1‐AS1 ORF2 peptide or ATMLP. Bars, 7 µm.

The enhanced green fluorescent protein (EGFP)‐N1‐ORF2 or EGFP‐N1 plasmids were transfected into A549 cells. Immunofluorescence analysis showed that the AFAP1‐AS1 ORF2 peptide colocalized with mitochondria based on mitochondrial‐specific COX‐4 antibody staining (**Figure** **4**A–C). To investigate which domains interact with mitochondria, we generated AFAP1‐AS1 ORF2 peptide truncation constructs with a C‐terminal EGFP‐tag and expressed them in HSAEC1‐KT cells and 293T cells. No other regions of the AFAP1‐AS1 ORF2 peptide except constructs containing amino acid residues 14–71 (base 2542–2716 in *AFAP1‐AS1*) retained the ability to interact with mitochondria (Figure 4D). We detected autophagy signals with an LC3B‐mCherry‐EGFP system, and the results showed that the AFAP1‐AS1 ORF2 peptide could significantly inhibit autophagy in A549 cells after 24 h serum starvation, while no significant change in the number of autophagosomes was detected (Figure 4E,F). This result indicated that the cells had undergone primary autophagy, but the autophagosomes may not have fused with lysosomes, resulting in GFP not being degraded. Staining and colocalization experiments of mitochondria and lysosomes confirmed that the AFAP1‐AS1 ORF2 peptide inhibited autolysosome formation. In cells overexpressing the AFAP1‐AS1 ORF2 peptide, neither radiation, serum starvation, nor rapamycin induced autolysosome formation (Figure 4G). Thus, the AFAP1‐AS1 ORF2 peptide could inhibit the formation of autolysosomes to prevent the degradation of the cell contents and subsequent cell death. Cell clonogenic formation studies (Figure S5A,B, Supporting Information) and proliferation experiments (Figure S5C,D, Supporting Information) also confirmed that the absence of the AFAP1‐AS1 peptide could significantly increase the radiosensitivity of cells. Thus, the AFAP1‐AS1 peptide could inhibit the formation of autolysosomes to prevent the degradation of the cell contents and subsequent cell death, thereby increasing the radioresistance of cells. For clarity, the AFAP1‐AS1 ORF2 peptide, due to its location, would be referred to as the lncRNA *AFAP1‐AS1 translated mitochondrial‐localized peptide* (ATMLP).

### 2.5 AFAP1‐AS1 ORF2 Peptide (ATMLP) Inhibits Autolysosome Formation by Binding to and Trapping NIPSNAP1 Protein to the Mitochondrial Inner Membrane

**Figure 5:**
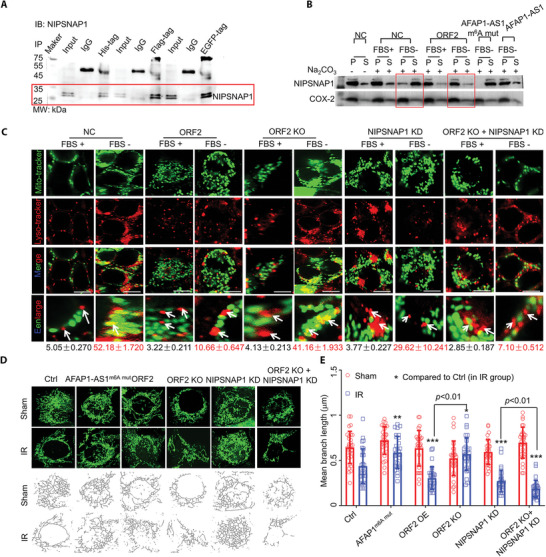
The AFAP1‐AS1 ORF2 peptide interacts with the NIPSNAP1 protein and inhibits autophagolysosome formation. A) Proteins that interact with the AFAP1‐AS1 ORF2 peptide were identified by coimmunoprecipitation (CoIP). AFAP1‐AS1 ORF2 peptide‐GFP/Flag/His plasmid was transfected into A549 cells, AFAP1‐AS1 ORF2 peptide‐GFP/Flag/His complexes were coimmunoprecipitated using anti‐GFP/Flag/His antibody, and NIPSNAP1 was detected. B) Mitochondrial fractions from A549 cells were incubated in mitochondrial buffer alone or mitochondrial buffer containing Na_2_CO_3_ (pH 11.5) and centrifuged at 16 000 × *g* for 15 min. Pellet (P) and supernatant (S) fractions were immunoblotted. C) A549 cells were transfected with the indicated constructs, and the autophagolysosome formation rate was measured. The white arrows indicate lysosomes and mitochondria that have or have not been positioned. D,E) A549 cells were transfected with the indicated constructs, and the mitochondrial morphology was imaged by confocal microscopy (D up). Mitochondrial mean branch length and mean network size were analyzed by MINA in ImageJ (D down). Data are represented as the means ± SEMs. ORF2 in the figures depicts ATMLP. Bars, 7 µm.

ATMLP lacks homology with other known proteins. To further investigate the mechanism by which ATMLP acts in cancer progression, proteins that may interact with ATMLP were identified. Furthermore, to more accurately distinguish the proteins that interact with ATMLP, we constructed His‐, Flag‐, and EGFP‐tagged ATMLP fusion proteins. Proteins were identified by the coimmunoprecipitation (CoIP) method, and the top 25 intersecting proteins were listed in Table S1 (Supporting Information). Protein–protein interaction assays combined with GO annotation showed that most of the proteins that interacted with ATMLP were involved in mitochondrial autophagy, cytoskeleton dynamics regulation, the mitochondrial inner membrane, etc. (Figure S6, Supporting Information), suggesting that ATMLP might regulate cellular mitochondrial‐mediated autophagy. NIPSNAP homolog 1 (NIPSNAP1), which hit the most fingerprint peptide, was one of the top 25 proteins found to interact with ATMLP. We further confirmed the interaction between ATMLP and NIPSNAP1 with a co‐IP assay (**Figure** **5**A). To clarify the positioning of NIPSNAP1 on the mitochondrial membrane, alkaline Na_2_CO_3_ extraction was used to isolate the outer mitochondrial membrane. The rinsed mitochondria were then used to collect other proteins. The results showed that NIPSNAP1 was extracted into the supernatant in untreated cells in serum‐free medium but was partly retained in the pellet in cells overexpressing ATMLP or lncRNA *AFAP1‐AS1*. However, this phenomenon was reversed by the lncRNA *AFAP1‐AS1* m^6^A mutation (Figure 5B). These results demonstrate that ATMLP is closely related to NIPSNAP1‐mediated mitochondrial membrane transport, in contrast to the inner mitochondrial membrane protein cytochrome oxidase subunit II (COX‐2) (Figure 5B). We subsequently observed that overexpression of ATMLP inhibited the formation of autolysosomes. Moreover, this phenomenon disappeared with *Atmlp* knockout (Figure 5C). Corroborately, the knockdown of NIPSNAP1 also led to the inability to form autophagolysosomes. It is more certain that the knockdown of NIPSNAP1*1* could significantly alleviate the loss of autophagolysosomes caused by *Atmlp* knockout (Figure 5C). We then examined the consequences of the inability of cells to complete the autophagy process. The results showed that after radiation injury, the mitochondrial morphology and the mean branch length in the control group and the *AFAP1‐AS1* m^6^A mut group did not change significantly, but the mean branch length of the mitochondria in the ATMLP and NIPSNAP1 KD groups was significantly reduced. *Atmlp* knockout could resist the shortening of mitochondria caused by radiation damage, but this effect disappeared with the knockdown of NIPSNAP1 (Figure 5D,E). These data show that the mitochondria in the ATMLP‐expressing cells and in NIPSNAP1 KD cells were not completely cleared after being damaged and may continue to divide with mitochondrial damage.

### 2.6 ATMLP Promotes Malignant Transformation of Epithelial Cells and Tumorigenesis

**Figure 6:**
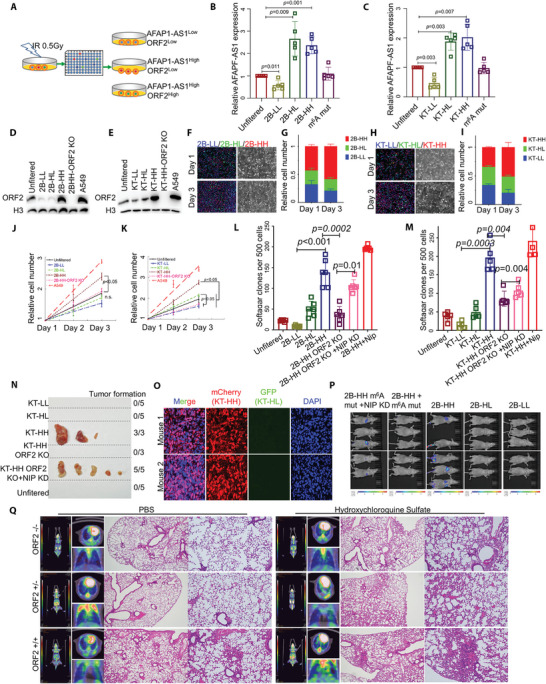
AFAP1‐AS1 ORF2 peptide, not lncRNA *AFAP1‐AS1*, promotes the malignant transformation of epithelial cells and the occurrence of lung cancer in situ in mice. A) Schematic of cell sorting conditions. A total of 3.5 Gy low‐dose ionizing radiation was given to HSAEC1‐KT (KT in short) and BEAS‐2B (2B in short) cells. By collecting single‐cell clones, low *AFAP1‐AS1* expression and low AFAP1‐AS1 ORF2 peptide expression cells (LL), high *AFAP1‐AS1* expression and low AFAP1‐AS1 ORF2 peptide expression cells (HL), and high *AFAP1‐AS1* expression and high AFAP1‐AS1 ORF2 peptide expression cells were identified for follow‐up experiments. B–E) *AFAP1‐AS1* RNA and ATMLP were verified with qPCR and western blot assays. F–I) The KT/2B‐LL (unlabeled), KT/2B‐HL (GFP‐labelled), and KT/2B‐HH (mCherry‐labelled) cell lines were seeded into Petri dishes in equal proportions. After 48 h, the distribution of fluorescent cells was counted. J,K) Detection of the proliferation rate of sorted cell lines: Unsorted cells and a tumor cell line were used as control groups. L,M) Soft agar sphere formation rate of sorted cell lines where unsorted cells and a tumor cell line were used as control groups. N) KT‐LL, KT‐HL, KT‐HH, KT‐HH with m^6^A mutant, KT‐HH with NIPSNAP1 KD and unfiltered cells were injected subcutaneously into the backs of mice. Tumor formation was evaluated after 120 days (*n* = 3 or 5). O) Equal numbers (1×10^5^) of the KT/‐LL (unlabeled), KT‐HL (GFP‐labeled), and KT‐HH (mCherry‐labeled) cell lines were injected subcutaneously into the backs of mice. Frozen sections were used to observe the distribution of fluorescent cells in the xenografts (*n* = 2). (P) After the luciferase‐labeled cells were injected into the tail vein, the distribution of the cells in the body and the intensity of the fluorescence signal were observed on day 90 by the small animal imaging system. Q) *AFAP1‐AS1 ORF2* KI or WT mice were irradiated with 2 Gy ionizing radiation and then injected with hydroxychloroquine or PBS. After 16 weeks, the uptake of ^18^F‐FDG in the lungs was checked by PET‐CT. ORF2 in the figures indicates ATMLP.

To study the main contribution of lncRNA *AFAP1‐AS1* and ATMLP to cell proliferation and malignant transformation, we irradiated immortalized, nontumorigenic human lung epithelial HASEC1‐KT (KT) and BEAS‐2B (2B) cells with a low 0.5 Gy dose of X‐rays 7 times every other day for a total dose of 3.5 Gy. The resuspended cells were seeded into 96‐well plates to form single cell clones. To elucidate the role of *AFAP1‐AS1* in promoting neoplastic transformation, we determined the ratio of *AFAP1‐AS1* to ATMLP in the cloned cell lines and divided them into three groups: *AFAP1‐AS1* low expression and ATMLP low expression (LL), *AFAP1‐AS1* high expression and ATMLP low expression (HL) and *AFAP1‐AS1* high expression and ATMLP high expression (HH) (**Figure** **6**A), which were verified by RT‐PCR and WB methods (Figure 6B–E). The three cell types with differential *AFAP1‐AS1* and ATMLP expressing different fluorescent labels were then replated into petri dishes and fixed on the first and the third day, and the proportion of each population was counted. HH cells proliferated the fastest (Figure 6F–I). The proliferation experiment also proved that the growth rate of HH cells was significantly faster than that of LL and HL cells, and this difference could be rescued by ATMLP knockout in HH cells (Figure 6J,K). To study the effect of *AFAP1‐AS1* and ATMLP on the malignant transformation of epithelial cells, we used a soft agar anchorage independence assay. The results showed that the anchorage‐independent growth of the HH group cells was significantly higher than that of the LL and HL cells, and the difference could be rescued by ATMLP knockout in HH cells. NIPSNAP1 knockdown also weakly increased the soft agar growth efficiency, whereas the growth of NIPSNAP1 knockdown among HH cells was the highest (Figure 6L,M). Xenograft experiments confirmed that control cells, KT‐LL, KT‐HL, and KT‐HH cells that all had ATMLP knocked out failed to form tumors, but KT‐HH cells, including KT‐HH cells with ATMLP knocked out plus NIPSNAP1 knocked down, were tumorigenic in nude mice (Figure 6N). The KT‐LL, KT‐HL, and KT‐HH cell lines with different fluorescent labels were subcutaneously injected into nude mice. After the xenografts were formed, the tumor slices were fixed, and the distribution of fluorescent signals was observed. Only KT‐HH cells labelled with mCherry grew into xenografts (Figure 6O). Lung colonization and tumor formation experiments with cells injected into the tail vein confirmed that 2B‐HH cells, including 2B‐HH cells in which NIPSNAP1 was knocked out, could form tumors in the lungs (Figure 6P). To verify this phenomenon in mice, we constructed mice with ATMLP knock‐in in mouse lung epithelial cells. We irradiated the lungs of mice with a single 2 Gy, and injected the autophagy inhibitor hydroxychloroquine into the designated group of mice. After 16 weeks, radiation treatment alone did not cause F^18^‐FDG uptake in the lungs of mice except for ATMLP KI homozygous mice. However, in ATMLP KI homozygous and heterozygous mice, the uptake of F^18^‐FDG in the lungs of mice was significantly increased after radiation treatment when combined with hydroxychloroquine injection. This also shows that ATMLP KI combined with autophagy inhibition could induce lung cancer in mice (Figure 6Q). These results indicate that the overexpression of ATMLP, not lncRNA *AFAP1‐AS1*, will promote the occurrence and progression of tumors, and the probability of this occurrence will increase as autophagy is inhibited.

### 2.7 ATMLP can be a Serum Marker for NSCLC Detection

**Figure 7:**
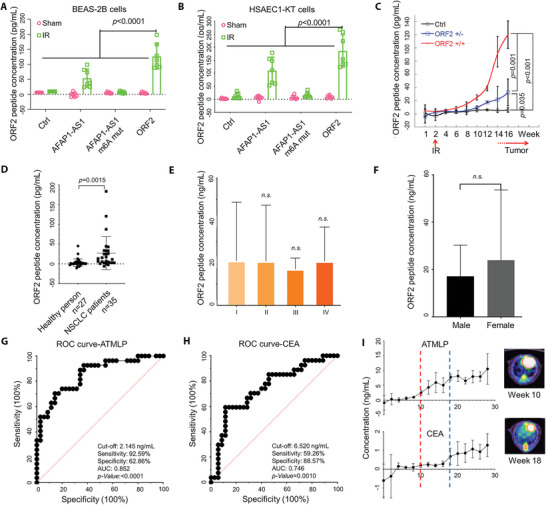
ATMLP can be used as a serum biomarker to detect lung cancer. A,B) The concentration of ATMLP in the cell supernatant after 2 Gy irradiation exposure was detected by ELISA. C) The concentration of ATMLP in mouse serum (*n* = 5). D–F) The concentration of ATMLP in the serum of healthy people (*n* = 27) and NSCLC patients (*n* = 35). ORF2 in the figures indicates ATMLP. G–H) ROC analysis of ATMLP and CEA in NSCLC patients. I) The concentrations of ATMLP and CEA in mouse serum (*n* = 4).

Upon irradiation with a sublethal dose of ionizing radiation, part of the mitochondria is likely to be damaged. Mitophagy provides a pathway whereby damaged mitochondria are cleared to maintain the normal survival of irradiated cells. However, if there is a large amount of ATMLP, which inhibits the fusion of lysosomes and autophagosomes on the mitochondria, it will prevent cells from undergoing autophagy and lead to two possible outcomes. The first is that cells continue to divide with damage, which may be a cause of tumors due to elevated oxidative stress. Another result is that the cells die. In the latter case, we detected ATMLP in the culture medium or serum other than the broken cells. As shown in **Figure** **7**, after irradiation, in HSAEC1‐KT and BEAS‐2B cells, no significant ATMLP was detected in the supernatant of the control group. However, the two groups overexpressing ATMLP and *AFAP1‐AS1* showed a strong ATMLP signal, which could be rescued by AFAP1‐AS1 m^6^A methylation site mutation (Figure 7A,B). Our previous data (Figure 6) confirmed that overexpression of ATMLP accelerated the occurrence of lung cancer caused by radiation. We next determined changes in ATMLP expression in serum during the neoplastic transformation process. After a week of acclimation, mice were given a 2 Gy dose of X‐rays to the lung using a CT‐guided small animal irradiator, 50 µL of blood was collected through the tail vein every two weeks, and the serum was analyzed by ELISA. The results showed that the expression of ATMLP in the serum of homozygous mice with lung‐specific ATMLP knock‐in as well as in heterozygous mice was significantly increased over time. However, no significant changes were observed in the control group mice (Figure 7C). These results show that with the occurrence and progression of lung cancer, the concentration of ATMLP in the serum will always increase. For further verification, we collected blood from 35 healthy donors and 27 NSCLC patients and found that the ATMLP in the serum of NSCLC patients was significantly higher than that of healthy people, while tumor TNM stage or sex did not affect the concentration of ATMLP (Figure 7D–F). To determine the underlying biomarkers driving the predictivity of the ensemble and characterize the discriminatory sensitivity and specificity of ATMLP, we subjected serum carcinoenbryonic antigen (CEA) and ATMLP serum concentrations to receiver operating characteristic (ROC) curve analysis. ROC curve analysis indicated that ATMLP had higher sensitivity and specificity than the classical biomarker ECA. Compared to the limited predictive power of CEA for early detection (AUC = 0.746), the AUC of ATMLP was 0.852 (*n* = 62, Table S2, Supporting Information; Figure 7G,H). This observation underscored the value of using ATMLP for early NSCLC detection. Next, we used the in situ lung cancer model induced by urethane to detect the time when ATMLP and CEA could be detected. The results showed that ATMLP could be significantly detected in serum at week 10 after urethane induction, while CEA needed 18 weeks. PET CT results showed that at the 10th week, there was little FDG uptake in the lungs. However, at the 18th week, significant FDG signals appeared in the lungs (Figure 7I). The results showed that ATMLP could predict the occurrence of lung cancer before PET‐CT imaging. Our findings demonstrated the potential status of ATMLP as a diagnostic serum biomarker of NSCLC.

## 3 Discussion

**Figure 8:**
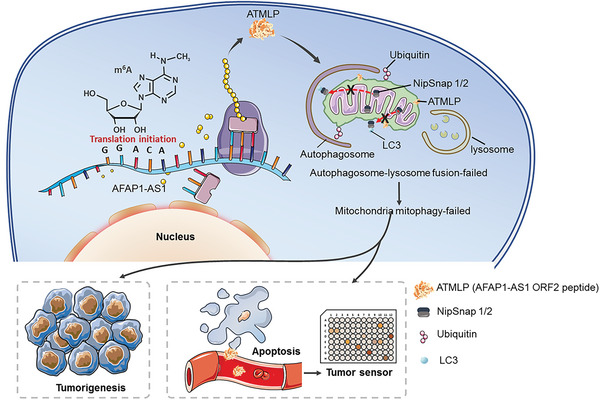
Working model for ATMLP function.

Micropeptides have traditionally been ignored due to their small size, but they are gaining increasing attention due to their critical roles in many essential biological activities. For example, a putative muscle‐specific lncRNA encodes a peptide named DWORF, which is localized to the sarcoplasmic reticulum (SR) membrane and enhances SERCA activity by displacing its inhibitors phospholamban, sarcolipin and myoregulin.^[^ ^14^ ^]^ Herein, our results demonstrate that a small peptide located on mitochondria could increase the viability of tumor cells by enhancing incomplete cell mitophagy. While lncRNAs themselves or their translated peptides are known to play an important role in various aspects of cell growth and differentiation, including carcinogenesis, our discovery of the lncRNA *AFAP1‐AS1* translation axis now implicates N^6^‐methyladenosine in their 5′‐UTR as a nodal regulator of peptide translation, providing an important link between lncRNAs and their translated peptide. However, we are not sure if this applies to other lncRNAs that can translate peptides (**Figure** **8**).

Recent advances in deep sequencing technologies have led to the identification of a large number of previously unknown transcripts. The vast majority (>99%) of these transcripts are considered lncRNAs, which do not appear to be translated into proteins given the lack of obvious long protein‐coding ORFs and clear homologues in other organisms.^[^ ^24^ ^]^ Accumulating evidence has shown that lncRNAs can regulate the physiological activities of cells by encoding polypeptides. Huang et al. discovered that the lncRNA *HOXB‐AS3* encodes a conserved 90 amino acid peptide that suppresses colon cancer (CRC) growth, indicating that its loss is a critical oncogenic event in CRC metabolic reprogramming.^[^ ^11^ ^]^ However, why lncRNAs translate peptides has confused many people. Eukaryotic mRNAs can be translated in both cap‐dependent and cap‐independent modes, although the mechanisms of translation initiation that do not require the 5′ cap and eIF4E are poorly understood. Meyer et al.^[^ ^25^ ^]^ showed that m^6^A residues within the 5′‐UTR can act as a m^6^A‐induced ribosome engagement site (MIRES), which promotes cap‐independent translation of mRNA. They found that a single m^6^A is sufficient to induce cap‐independent translation. The significance of 5′‐UTR m^6^A residues was further observed in both ribosome profiling datasets and in individual cellular mRNAs in conditions where cap‐dependent translation was suppressed. These results point to selective recognition of 5′‐UTR m^6^A as a mechanism for mRNAs to bypass the cap requirement for translation and suggest a potential role for this class of m^6^A residues in mediating translational responses induced in diverse cellular stress states. Our results confirmed that lncRNA *AFAP1‐AS1* could also translate proteins under the m^6^A site by making point mutations at the 1313 adenine locus site of *AFAP1‐AS1*, but we need more evidence to prove whether this translation regulation occurs in a cap‐independent manner.

Despite concerted efforts over the last few decades, the exact role of autophagy in cellular radiation responses has remained controversial. Two schools of thought exist: one suggests that autophagy is a cell survival phenomenon, while the other suggests that autophagy is a type II programmed cell death mechanism that helps in the removal of affected cells. Current understanding suggests that the type, extent, and time of stress are important determinants of the fate of a cell following autophagy induction.^[^ ^26^ ^]^ It is well established that radiation exposure leads to extensive mitochondrial biogenesis, thereby providing additional support for cell survival.^[^ ^27^ ^]^ However, under conditions of extensive mitochondrial damage, the cell employs mitophagy to eliminate damaged and dysfunctional mitochondria. Most studies linking radiation with autophagy have been performed on cancer patients undergoing radiotherapy. Elevated levels of autophagy have been found to be associated with both chemoresistance and radioresistance in various cancer types.^[^ ^28^ ^]^ Clinical trials combining chemotherapeutic agents with autophagy inhibitors such as chloroquine (CQ), hydroxychloroquine (HCQ) and 3‐methyladenine (3‐MA) provide survival benefits and increased life span in patients with breast cancer, myeloma, prostate cancer, and several other advanced tumors.^[^ ^29^ ^]^ In contrast to the role of autophagy in radioresistance, there is evidence that autophagy can also promote cell death.^[^ ^30^ ^]^ Various tumor suppressors have been shown to induce high levels of autophagy.^[^ ^31^ ^]^ For instance, loss of Beclin1 gene function has been associated with various solid tumors, including breast, ovarian and prostate tumors. Similarly, combined treatment with Akt inhibitors and radiation has been shown to induce autophagy in numerous carcinoma conditions, thus enhancing the radiosensitization of cancer cells.^[^ ^30^ ^]^ Molecular mechanisms through which autophagy helps tumor suppression are poorly understood. In the most well understood mechanism, autophagy degrades damaged and mutated cells that may otherwise gain oncogenic properties.^[^ ^32^ ^]^ Autophagy is an evolutionarily conserved catabolic process that delivers cellular constituents, including damaged or superfluous organelles and long‐lived proteins, to lysosomes for degradation and recycling, thereby regulating cellular homeostasis and inhibiting cancer formation. Therefore, we believe that ATMLP inhibits autophagy and causes normal cells to escape autophagy after being stressed, which is conducive to the transition of normal cells into tumor cells. However, overexpression of the *AFAP1‐AS1* peptide in tumor cells is not necessarily beneficial to radiation stress in tumors. Tumor cells may evolve unfavorable factors themselves, and if so, there are presumably other pathways involved in this aspect of regulation. Therefore, our research results show that the *AFAP1‐AS1* peptide does not affect the formation of autophagosomes but inhibits the formation of autophagolysosomes. Collectively, the *AFAP1‐AS1* peptide induces the earliest stage of autophagy, but the process fails to come to fruition. Our evidence proves that autophagy has not been strictly enforced. Mitochondria have not been swallowed in large numbers. A large number of mitochondria are consumed by autophagy, which causes the loss of mitochondrial homeostasis, which may also be an important cause of tumorigenesis. However, how normal cells obtain abnormal expression of ATMLP after radiation stress is the focus of our next study.

In summary, we show that a lncRNA can be translated by engaging m^6^A methylation. In this way, the lncRNA *AFAP1‐AS1* can translate its ATMLP peptide, which acts as an oncogene. Our results provide a roadmap where this can be exploited in cancer treatment based on both the properties of lncRNAs and their protein‐coding ability. Furthermore, the findings provide a clearer understanding of the process of tumorigenesis with translational potential.

## 4 Experimental Section

### Cell Lines

Human nonsmall cell lung carcinoma A549 and Calu‐1, immortalized human bronchial epithelial cells (Beas‐2B), human small airway epithelial cells (HSAEC1‐KT) and human embryonic kidney 293T cells (HEK293T) were obtained from the American Type Culture Collection (Rockville, MD, USA). All cell lines were used within 10–20 passages according to the ATCC recommendation. Cell lines were routinely tested for mycoplasma contamination using a mycoplasma detection kit (Beyotime, Guangzhou, China). All cell lines were grown in Dulbecco's modified Eagle's medium (DMEM) supplemented with 10% fetal bovine serum and maintained in a 37 °C, 5% CO_2_ incubator if not specified.

### Clinical Specimens

NSCLC tissue microarrays were purchased from Shanghai Superbiotek Company (LUC1601 and LUC1602). Microarrays were immunostained with AFAP1‐AS1 peptide antibody (Abclonal, Wuhan, China). Patient pathological information was also provided by Shanghai Biochip Company. NSCLC tissues and matched adjacent normal tissues were reviewed and approved by the Department of Pathology of the Second Affiliated Hospital of Soochow University. All staining was assessed by a quantitative imaging method, and the percentage of immunostaining and the staining intensity were recorded. The H‐score was calculated using the following formula: H‐score = (percentage of cells of weak intensity × 1) + (percentage of cells of moderate intensity × 2) + (percentage of cells of strong intensity × 3). The serum of NSCLC patients was obtained from the Second Affiliated Hospital of Soochow University. Information can be seen in Table S2 (Supporting Information). The use of human tissues and serum was approved by the Ethics Committee of Soochow University (approval number SUDA20211117A01), and written consent was obtained from all participants (or their next of kin) who provided tissue/serum.

### Animal Experiment

BEAS‐2B and HSAEC1‐KT cell lines, including their genome editing variants, were used. Briefly, 1×10^6^ cells were injected subcutaneously into the flanks of 8‐week‐old nude mice (*n* = 5). After 3 months, the xenografted tumors were dissected, categorized and photographed. In vivo lung metastasis was assessed by injection of 5×10^5^ luciferase‐labelled cells into the tail veins of NOD/SCID mice (*n* = 5). After 60 days, the imaging of the cells in the lungs was observed by an IVIS spectrum small animal imager (PerkinElmer). Mouse tumor tissues or lungs were fixed with 4% formalin (V/V). Tissues were embedded in paraffin after dehydration, and 5 µm sections were stained with hematoxylin–eosin and photographed (Hamamatsu Photonics). All mice were cared for in the SPF Animal Laboratory, Soochow University, under the approval of the Institutional Review Board or Animal Care and Use Committee.

### RNA‐Seq

Collected total RNA samples from cells exposed to 2 Gy X‐ray radiation were subjected to high‐throughput sequencing. Agilent Human lncRNA Microarray 2018 Version (4×180 k, Design ID: 085630) was used in this experiment, and data analysis of the samples was completed by OE‐Biotech. Feature Extraction software (version 10.7.1.1, Agilent Technologies) was used to analyze array images to obtain raw data. Genespring (version 13.1, Agilent Technologies) was employed to finish the basic analysis with the raw data. Differentially expressed genes or lncRNAs were then identified through fold change as well as P value calculated with t test. The threshold set for up‐ and downregulated genes was a fold change > 2 and a *p* value < 0.01. Each lncRNA was identified as a cis‐regulated mRNA when the mRNA loci were within 300 k windows up‐ and downstream of the given lncRNA or the Pearson correlation of lncRNA‒mRNA expression was significant (*P* value of correlation <0.05). The lncRNAs and their cis‐regulated mRNAs were transcription factors by calculating the significance of GENE enrichment in each transcription factor.

### qRT‐PCR

Total RNA was extracted from all cell lines used in this study with TRIzol total RNA isolation reagent (Life Technologies). The RNA levels of *AFAP1‐AS1* and *Gapdh* were detected using qRT‐PCR. The *AFAP1‐AS1* primers were as follows: forwards, 5‐GGGGTAACTCAAAAAGCCTGT‐3, reverse, 5‐GGGGGTGTAGCAGCAATTCA‐3. The *Gapdh* primers were as follows: forwards, 5‐ ACAACTTTGGTATCGTGGAAGG′‐3, reverse, 5‐ GCCATCACGCCACAGTTTC‐3.

### Gene Silencing, Overexpression, and Reporter Plasmids

Cells (1 × 10^5^) were transfected with 1 × 10^6^ shRNA lentivirus particles (Sangon, Shanghai, China) for 24 h. Control shRNA and GFP lentivirus particles were also used according to the manufacturer's instructions. Cells were subsequently screened using MEM containing 2 × 10^−6^ m puromycin (Invitrogen) and double‐checked by RT‐PCR. Adenovirus particles were used for gene overexpression (Sangon). pcDNA 3.1‐ORF2, EGFP‐C1/EGFP‐N1‐ORF2, and pAAV‐U6‐PolyA recombinant plasmids were also used in some experiments. Cells in a 12‐well plate were cotransfected with 300 ng DNA using Lipofectamine 3000. All siRNAs were purchased from Sangon Biotech (Shanghai). The target sequences for NIPSNAP1 were as follows: siRNA1 CCAGGAACCAUGAUCGAGU, siRNA2 CGUAACAGGAACUCGGAAG.

### Identification of Conserved Small Open Reading Frames (ORFs)

Transcripts were extracted and combined with transcripts annotated as lncRNAs in the UCSC database. ORF finder in NCBI searches for open reading frames (ORFs) in the target lncRNA transcripts with ATG start codons (https://www.ncbi.nlm.nih.gov/orffinder/). The newly sequenced lncRNAs were examined for potential protein‐encoding segments, and predicted proteins were verified using the newly developed SMART BLAST. PhyloCSF was used to calculate conservation scores for potential ORFs in all three frames on both strands.^[^ ^23^ ^]^ Only the highest scoring ORF for the transcript was reported. The ribosome profiling data retrieved in GWIPS‐viz Genome Browser revealed that *AFAP1‐AS1* RNA was occupied by ribosomes.^[^ ^33^ ^]^

### Anti‐ATMLP Antibody Preparation

Peptide synthesis and anti‐ATMLP antibody preparation were performed by ABclonal Technology Co., Ltd. (Wuhan). Through epitope prediction, the peptide sequence GQSRMKSPVSNTN‐C was selected to be synthesized and used to immunize rabbits in generating antibodies. Polyclonal and monoclonal antibodies against ATMLP were obtained from inoculated rabbits. Antibodies were purified using affinity chromatography on columns containing the corresponding peptides.

### CRISPR/Cas9 Genome Point Mutation Editing in Cells

*AFAP1‐AS1* 1311–1315 m^6^A mutated cells were generated using CRISPR/Cas9 technology. pSpCas9(BB)‐2A‐Puro (PX459) V2.0 was a gift from Feng Zhang (Addgene plasmid # 62988; http://n2t.net/addgene:62988; RRID:Addgene_62988).^[^ ^34^ ^]^ Briefly, the CHOPCHOP web tool was used to design a set of three single guide RNA (sgRNA) molecules targeting exon 2 in the *AFAP1‐AS1* gene. Individual sgRNAs were mixed with Cas9 plasmid and donor DNA. For vectors with the puromycin resistance gene, cells were treated with 2 µg mL^−1^ puromycin 24 h posttransfection for 36 h. Single cells were then sorted and plated into 96‐well plates. For vectors with the EGFP gene, EGFP‐positive cells were sorted by FACS and plated into 96‐well plates 48 h post‐transfection. Single colonies were then expanded and screened by immunoblotting. The AFAP1‐AS1 peptide was validated by immunoblotting. Synthesis and single‐cell screening were performed by Riobio. To knock out *ORF2* in cells, the following gRNA pair flanking *ORF2*: gRNA‐1: GCGGCTATTGAAGTGAACGCCGG; gRNA‐2: TCACTTCAATAGCCGCTCGAAGG; gRNA‐3: AAAGGACCTATTGCTCACCA; gRNA‐4: AACGCCGGTATGAAGGGTGT was used. To knock out Nipsnap1 in cells, the following gRNA pair: gRNA‐1: ACTCCATGTGTCGCACCAGGGGG; gRNA‐2: AGTTGCCCACGAGTGAGCATGGG was used.

### Construction of TRICK Translation Biosensors

Construction of TRICK translation biosensors was performed as described.^[^ ^35^ ^]^ PP7 stem‐loops that could be translated by the ribosome were generated by gene synthesis by removing potential stop codons in all three reading frames. The cassette (405 nt) contained six copies of the PP7 stem‐loops that were positioned 40 nucleotides apart. The 6×PP7 stem‐loop cassette was inserted into the target mRNA sequence. After the stop codon, the 12×MS2 stem‐loop cassette was placed. Chimeric fusions of the PP7 coat protein (PCP) and MS2 coat protein (MCP) with fluorescent proteins were generated to label reporter mRNAs. An NLS was added to PCP, and this was fused to the N‐terminus of TagRFP to make NLS‐PCP‐RFP. A single chain tandem dimer of MCP, which also contained an NLS, was fused to the N‐terminus of EGFP to make NLS‐MCP‐EGFP. Upon export of the reporter RNA, the first round of translation displaces NLS‐PCP‐RFP from the transcript as the ribosome traverses the coding region that contains the PP7 stem‐loops. The NLS limits the concentration of free NLS‐PCP‐RFP in the cytoplasm, yielding translated RNAs that are labelled with only NLS‐MCP‐GFP bound to the stem‒loops in the 3′ UTR. Both fluorescent fusion proteins were cloned into the pFucci‐Blasti lentiviral vector.

### Western Blotting

Cells were suspended in lysis buffer (50 mM Tris‐HCl, pH 7.4, 150 × 10^−3^ m NaCl, 0.1% SDS, 1% Triton X‐100, 1 × 10^−3^ m EDTA, 5 × 10^−3^ m DTT, and protease inhibitor cocktail) and boiled. The cellular lysates were centrifuged at 13 000 rpm for 10 min. The protein concentration was determined by BCA assay (Beyotime, Hangzhou). Western blotting was performed using anti‐EGFP (1:10 000, CST) or anti‐ATMLP (1:500, Abclonal) antibodies. For the detection of NIPSNAP1 and Cox‐2, western blotting was performed using anti‐NIPSNAP1 (1:1000, Abcam) and Cox‐2 (1:1000, CST) antibodies.

### RNA m^6^A Individual Nucleotide Resolution Crosslinking and Immunoprecipitation (miCLIP)

miCLIP was performed using A549 cells as described^[^ ^25^ ^]^ with some modifications. Briefly, *AFAP1‐AS1* or its mutation sequence was transfected into cells with U6 or CMV promoter plasmid. Cells cultured in 100 mm diameter tissue culture dishes were washed with cold PBS. Cells were scraped after 400 µL of cell lysis buffer was added per plate. Cell lysates were directly diluted in 450 µL immunoprecipitation buffer (50 × 10^−3^ m TRIS pH 7.4, 100 × 10^−3^ m NaCl, 0.05% NP‐40) and incubated with 3 µg anti‐m^6^A (SYSY, 202003; Abcam) at 4 °C for 4 h, rotating head over tail. The cell lysate was irradiated once at 400 mJ cm^−2^ in the Stratalinker. After irradiation, the solution was transferred into tubes and incubated with 30 µL of Protein A/G beads (Thermo Scientific) for 2 h at 4 °C with rotation. Bead‐bound antibody‐RNA complexes were then treated with DNase and proteinase K (Invitrogen). Bead‐bound antibody‐RNA complexes were then recovered on a magnetic stand (Life Technologies) and washed twice with high‐salt buffer (50 × 10^−3^ m Tris pH 7.4, 1 m NaCl, 1 mM EDTA, 1% NP‐40, 0.1% SDS), twice with immunoprecipitation buffer, and twice with polynucleotide kinase (PNK) wash buffer (20 × 10^−3^ m Tris, 10 × 10^−3^ m MgCl_2_, 0.2% Tween 20). RNA was reverse‐transcribed (TAKARA). Amplify the cDNAs by RT‐PCR or sequencing. The primers for S1 were as follows: forwards‐GACCCTAAACTCCACAGTTCCCAAA, reverse‐AGATGCAGGAGGGCCAGGAGTGCTT; S2: forward‐AGGCGAGGTTCTCTTTTTCAAAGCC, reverse‐GGACTTTGTGCCTCAATGATCTGAT; S3: forward‐TTCATCAGATCATTGAGGCACAAAG, reverse‐TGAAGTCACAGAAACTGAGTTTGCA; and S4: forwards‐TGGCTGCAAACTCAGTTTCTGTGAC, reverse‐ACAGTGTTTCTTGGGGCTTGGAAAC.

### Subcellular Fractionation and Sodium Carbonate Extraction

Subcellular fractionation was performed with a Mitochondria Isolation Kit (Beyotime) according to the instruction manual. In brief, 10^7^ cells were resuspended in 1 mL of lysis buffer, incubated for 10 min at 4 °C, and centrifuged at 1000 × *g* for 10 min. The supernatant was transferred into a separate tube as the cytosolic fraction, while the pellet was resuspended in 1.5 mL of ice‐cold disruption buffer, rapidly passed through a 21 g needle 10 times to disrupt cells, and centrifuged at 1000 × *g* for 10 min at 4 °C. The pellet was saved as the nuclear fraction, while the supernatant was recentrifuged at 6000 × *g* for 10 min at 4 °C. The pellet obtained after centrifugation comprised the mitochondrial fraction, while the supernatant contained the microsomal fraction. For analysis of integral membrane proteins, the mitochondrial fraction was resuspended in mitochondrial buffer (210 × 10^−3^ m mannitol, 70 × 10^−3^ m sucrose, 10 × 10^−3^ m HEPES, 1 × 10^−3^ m EDTA, pH 7.5) or mitochondrial buffer containing freshly prepared 0.1 m Na_2_CO_3_ (pH 11.5) and incubated on ice for 30 min. The insoluble membrane fraction was centrifuged at 16 000 × *g* for 15 min.

### Mitochondrial Staining and Analysis of Mitophagy

Mitochondrial morphological analysis was performed with the MINA ImageJ macro tool.^[^ ^36^ ^]^ Mitochondria were stained with 100 × 10^−3^ m MitoTracker Deep Red FM (Beyotime, China) for 10 min at 37 °C. Lysosomes were stained with 50 × 10^−3^ m Lysotracker Red (Beyotime, China) for 10 min at 37 °C. Fluorescence images were acquired, and the number of colocalized spots of mitochondria and lysosomes was quantified using ImageJ software. At least 300 cells were scored for each experiment. For the mitophagy assay, the mCherry‐EGFP‐LC3B double fusion fluorescent protein system (Beyotime, China) was used to detect cell autophagy. A549 and Calu‐1 cells seeded in 3.5 cm dishes (or 24‐well plates for confocal microscopy) were treated with 10 × 10^−6^ m oligomycin or serum‐free culture for the indicated times. After cells were transfected with the mCherry‐GFP‐LC3B system, under nonautophagic conditions, mCherry‐GFP‐LC3B existed in the cytoplasm in the form of diffuse yellow fluorescence (superimposed effect of mCherry and GFP) when observed under the fluorescence microscope. In the case of autophagy, mCherry‐GFP‐LC3B aggregated on the autophagosome membrane and appeared in the form of yellow spots (LC3B dot or punctate); when the autophagosome fused with the lysosome, GFP fluorescence was quenched and appeared in the form of red spots. LysoTracker Red and MitoTracker Green (Beyotime. China) reporter were then used for visualizing acidified autolysosomes.

### Coimmunoprecipitation (CoIP) and Mass Spectrometry

CoIP was performed as previously described. A549 or HSAEC1‐KT cells were separately transfected with EGFP‐ATMLP, Flag‐ATMLP and 6HIS‐ATMLP, IP was carried out using mouse anti‐EGFP, anti‐Flag or anti‐HIS (Abcam) antibodies, and samples were collected using Dynabeads (Life Technologies). The taged ATMLP complexes were separated, and the gels were stained with bromol blue. Three independent experiments were performed. The differential gel bands and their corresponding negative gel bands were excised and subjected to digestion. The extracted protein mixtures were dissolved in buffer containing 0.1% formic acid and 2% acetonitrile (AcN) and analyzed using nano‐LC‒MS/MS (AB SCIEX TripleTOF 5600, USA) by PTM Bio (Hangzhou). Standard western blot procedures were performed on IP fractions using HRP‐conjugated GFP, FLAG, and HIS antibodies (Beyotime).

### AFAP1‐AS1 Peptide Indirect Enzyme‐Linked Immunosorbent Assay (ELISA)

The concentration of AFAP1‐AS1 peptide (ORF2 peptide) in the cell culture supernatant, mouse serum, and human serum of healthy people or NSCLC patients was detected with ELISA experiments. As a comparison, human intrinsic factor antibody (IFA) was applied as a positive control. Cell culture supernatant or serum was added to an enzyme‐linked plate with coating buffer for 24 h in 4 °C. They were washed three times with PBST and blocked with 10% BSA at 37 °C for 1 h. After three washes with PBST, samples were treated with an anti‐AFAP1‐AS1 peptide antibody (ABclonal) at a 1:100 dilution with PBS for 30 min at 37 °C. After a final four washes with PBST (PBS+1% Tween 20, pH = 7.5), all wells were treated with 100 µL TMB color developing solution and incubated at 37 °C in the dark for 15 min, and then 2 × 10^−3^ m sulfuric acid was added to stop the reaction. The Human CEA ELISA Kit (Abcam) and Mouse Carcinoembryonic Antigen (CEA) ELISA Kit (Cusabio) were used for serum CEA detection. All wells were examined with a microplate reader at a wavelength of 450 nm. The sensitivity and specificity of all biomarkers for lung cancer diagnosis were evaluated by receiver operating characteristic (ROC) curves and areas under the curves (AUCs) with 95% confidence intervals (CIs).

### Generation of AFAP1‐AS1 Knock‐In (KI) Mice

The AFAP1‐AS1‐ORF2‐PolyA gene fragment carrying the hSFTPC promoter was inserted into the H11 site of the C57BL/6JGpt mouse. According to the design plan, gRNA was designed, constructed and transcribed in vitro, and the homologous recombination vector (donor vector) was used to verify the correctness of the vector by sequencing. The gRNA sequence was CTGAGCCAACAGTGGTAGTA. The Cas9, gRNA, and donor vector samples were microinjected into fertilized mouse eggs on the C57BL/6JGpt background. The fertilized eggs that survived the injection were transplanted into pseudopregnant female mice to generate F0 offspring. The pups from the recipient mice were trimmed and numbered after 5–7 days, and genomic DNA was extracted for PCR amplification, sequencing and southern identification to confirm the genotype. After the positive F0 generation mice became sexually mature, they were mated with wild‐type background mice whose genotype was checked and confirmed as described above.

### Statistics

All experiments were independently repeated at least three times, and all data are presented as the mean ± standard error. Student's *t* tests were employed for statistical analysis, and a probability (*p*) value less than 0.05 was considered statistically significant.

### Data Availability

More detailed methods are available in the Supporting Information and Experimental Section. All relevant data supporting the findings of this study are available within the article and its Supporting Information or from the authors upon reasonable request.

## Conflict of Interest

The authors declare no conflict of interest.

## Supporting information

Supporting Information

## Data Availability

The data that support the findings of this study are available in the supplementary material of this article.
