# Supplementary material for: The Tumorigenic Effect of lncRNA AFAP1‐AS1 is Mediated by Translated Peptide ATMLP Under the Control of m6A Methylation
Source: Adv Sci (Weinh). 2023 Mar 4;10(13):2300314. doi: 10.1002/advs.202300314 (PMC10161021; doi:10.1002/advs.202300314)
Supplement: Supplementary file 1 — Supporting Information [file ADVS-10-2300314-s001.pdf]

ADVANCED SCIENCE
Open Access

Supporting Information

for *Adv. Sci.*, DOI 10.1002/advs.202300314

The Tumorigenic Effect of lncRNA AFAP1-AS1 is Mediated by Translated Peptide ATMLP Under the Control of m^{6}A Methylation

Hailong Pei, Yingchu Dai, Yongduo Yu, Jiaxin Tang, Zhifei Cao, Yongsheng Zhang, Bingyan Li, Jing Nie, Tom K. Hei* and Guangming Zhou**

Supporting Information

Title

The tumorigenic effect of lncRNA AFAP1-AS1 is mediated by translated peptide ATMLP under the control of m^6A methylation.

Hailong Pei, Yingchu Dai, Yongduo Yu, Jiaxin Tang, Zhifei Cao, Yongsheng Zhang, Bingyan Li, Jing Nie, Tom K. Hei*, and Guangming Zhou**

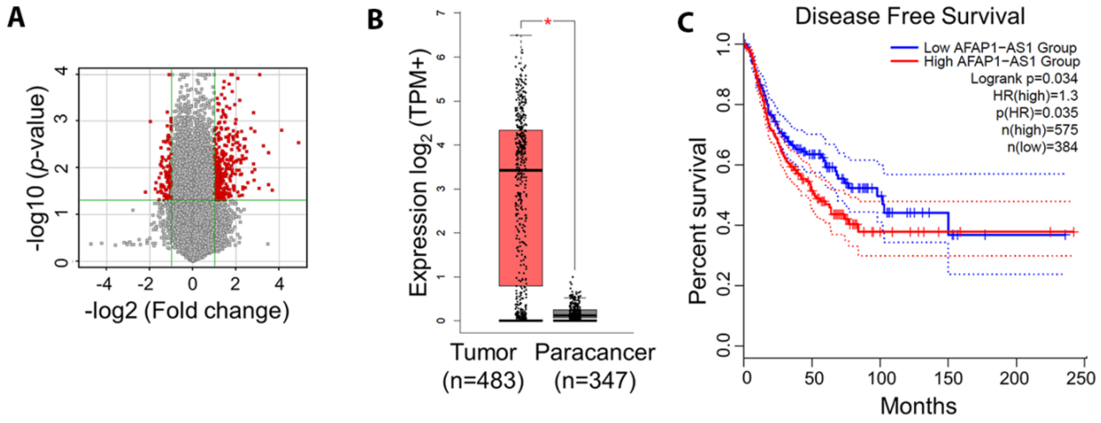

Figure S1. AFAP1-AS1 is related to radiation stress and tumorigenesis.

(**A**) Intersection expression profiles of differentially expressed lncRNAs in A549 cells induced by 2 Gy X-ray exposure. (**B**) *AFAP1-AS1* RNA levels compared between NSCLC tissues and corresponding paracancerous (P) tissues in the indicated cancers by database prediction. (**C**) Kaplan–Meier survival analysis of NSCLC patients according to *AFAP1-AS1* RNA expression ratios of cancer/paracancer tissues.

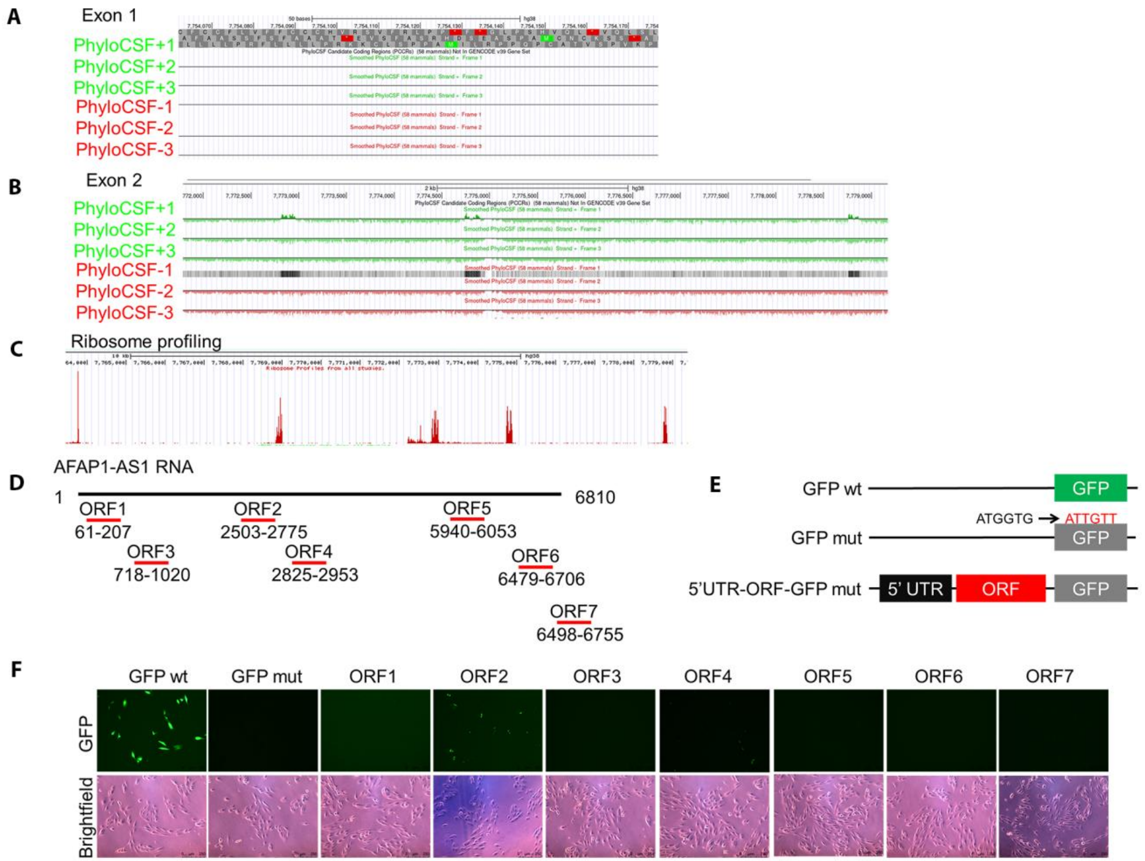

Figure S2. Potential open reading frame of *AFAP1-AS1*. (**A** and **B**) PhyloCSF score plot for the *AFAP1-AS1* exon 1 and exon 2 locus as seen in the UCSC genome browser using the PhyloCSF track hub. (**C**) The ribosome profiling data retrieved in the GWIPS-viz Genome Browser revealed that lncRNA *AFAP1-AS1* RNA was located in ribosomes. (**D**) Diagram of the ORFs in *AFAP1-AS1*. (**E**) Diagram of the GFP fusion constructs used

for transfection. (**F**) The indicated constructs were transfected into BEAS-2B cells for 24 h, and GFP fluorescence was observed as a surrogate marker for protein expression after 24hs.

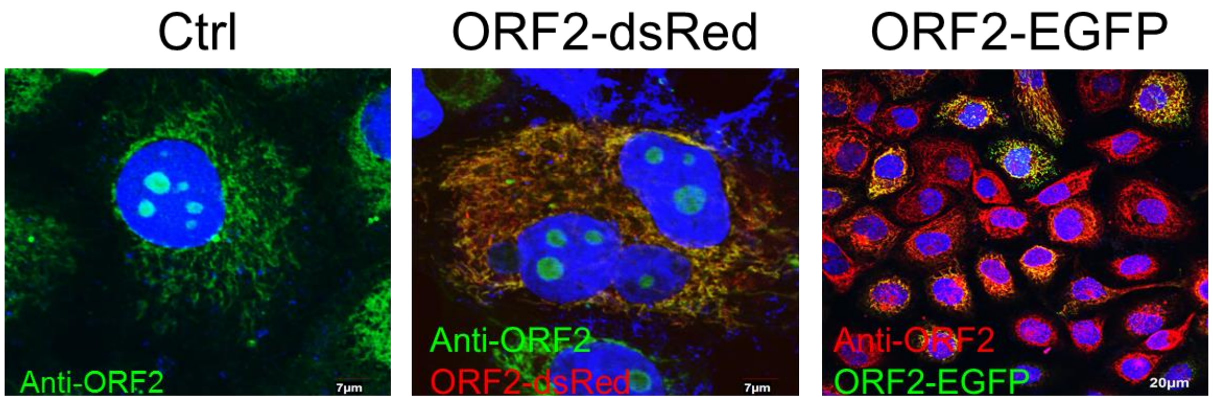

Figure S3. Immunofluorescence staining of cells. The indicated constructs were expressed in A549 cells, and the AFAP1-AS1 peptide fusion protein and ORF2 were determined. Bars: 7 µm or 20 µm.

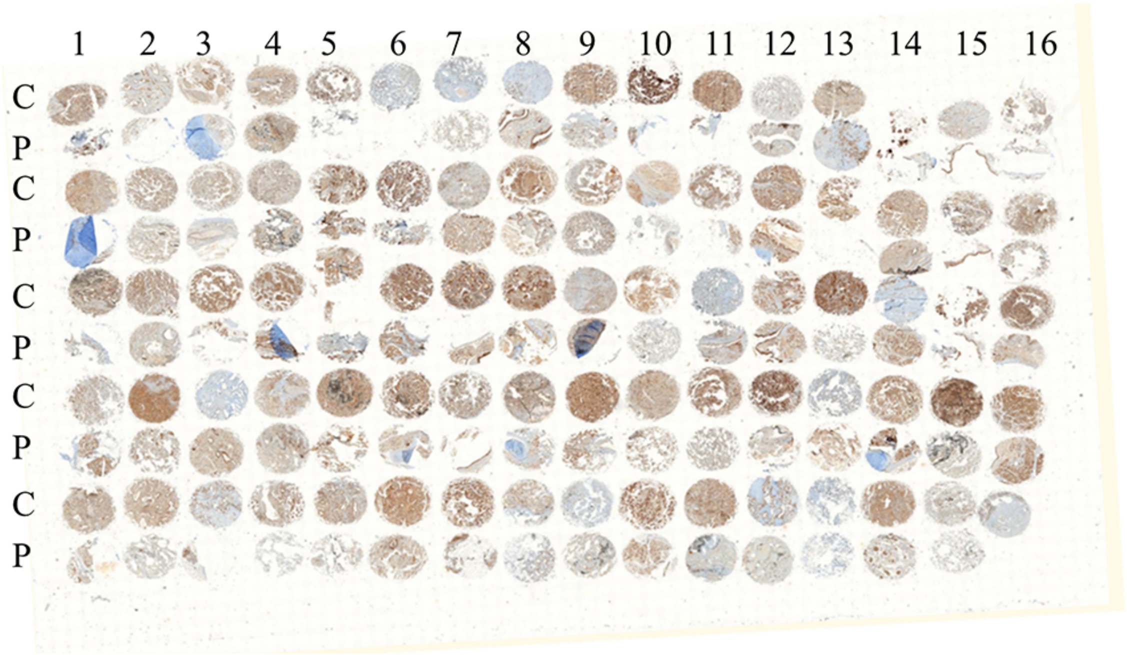

Figure S4. Representative IHC images of AFAP1-AS1 peptide expression in NSCLC tissues and corresponding paracancerous tissues. Microarray from Shanghai Biochip Company.

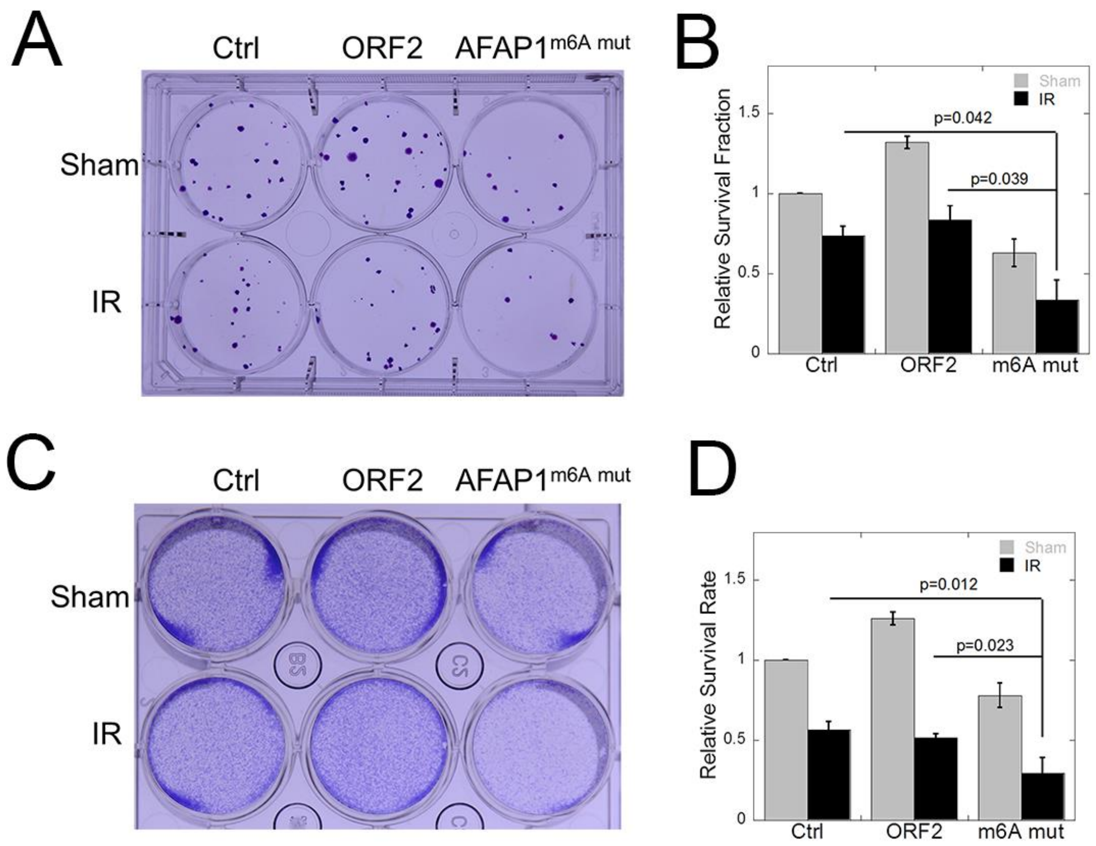

Figure S5. AFAP1-AS1 peptide, but not AFAP11-AS1 lncRNA, increases NSCLC cell radioresistance. (A, B) A549 cells were transfected with the indicated constructs, and their colony-forming abilities were measured after 2 weeks (n = 5). (C, D) A549 cells were transfected with the indicated constructs for the indicated times, and the number of cells was measured (n = 3). Data are represented as the means ± SEMs. ORF2 in the figures indicates the AFAP1-AS1 peptide.

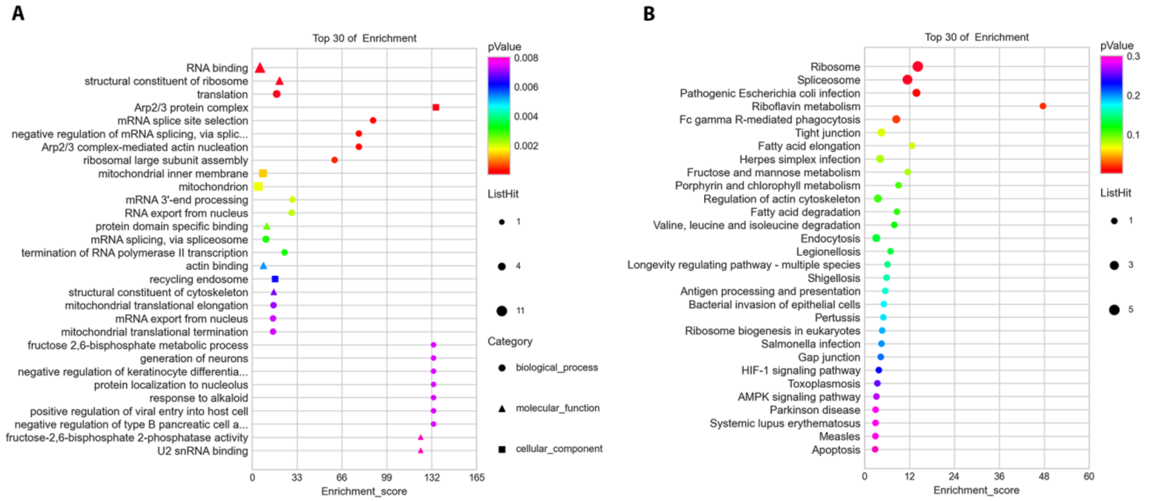

Figure S6. Pathway analysis of AFAP1-AS1 peptide-interacting proteins. (**A**) GO pathway analysis. (**B**) KEGG pathway analysis.

Table S1. Top 25 intersecting proteins engaging in specific interactions with the AFAP1-AS1 ORF2 peptide.

Protein names	Gene names	MW(kDa)	Sequence coverage (%)	Unique Peptides
Protein NipSnap homolog 1	NIPSNAP1	33.29	49.3	13
Tubulin alpha-1A chain	TUBA1A	50.1	41.46	11
Heat shock 70 kDa protein 1-like	HSPA1L	70.33	14.2	6
60S ribosomal protein L3	RPL3	46.08	10.92	5
GTP-binding nuclear protein Ran	RAN	24.41	11.11	5

39S ribosomal protein L11, mitochondrial	MRPL11	20.67	20.83	3
Keratin, type II cytoskeletal 5	KRT5	62.34	7.46	3
Serine/arginine-rich splicing factor 9	SRSF9	25.53	9.5	2
40S ribosomal protein S26	RPS26	13.01	13.4	2
28S ribosomal protein S2, mitochondrial	MRPS2	33.23	2.7	2
60S ribosomal protein L26	RPL26	17.25	10.34	2
Drebrin	DBN1	71.39	3.08	2
Actin-related protein 3	ACTR3	47.34	5.5	2
Trifunctional enzyme subunit beta, mitochondrial	HADHB	51.26	4.66	2
Actin-related protein 2/3 complex subunit 1B	ARPC1B	40.92	4.84	2
6-phosphofructo-2-kinase/fructose-2,6-bisphosphatase 3	PFKFB3	59.57	4.81	2
Flavin reductase (NADPH)	BLVRB	22.11	14.08	2
U2 small nuclear ribonucleoprotein A'	SNRPA1	28.4	9.02	1
E3 ubiquitin-protein ligase TRIM21	TRIM21	50.14	2.11	1
rRNA methyltransferase 3, mitochondrial	MRM3	46.99	2.62	1
Uncharacterized protein C1orf167	C1orf167	162.32	0.54	1
Ubiquitin-like modifier-activating enzyme 1	UBA1	117.77	1.04	1
Serine/arginine-rich splicing factor 6	SRSF6	39.56	2.03	1
Mitochondrial mRNA pseudouridine synthase TRUB2	TRUB2	36.67	3.93	1

Cofilin-1	CFL1	18.49	16.63	1
Plakophilin-2	PKP2	97.35	1.25	1

Table S2. Clinical characteristics of the study population.

Organizational coding	Gender	Age	Distant metastasis	Original organ	Pathogenic	Tumor size	T	N	M	AJCC Seventh Edition Clinical Staging
NSCLC0001	F	74	N	Y	Adenocarcinoma	1.8 cm	T1	N0	M0	Ⅰ A
NSCLC0002	M	63	N	Y	Adenocarcinoma	2.5 cm	T1	N0	M0	Ⅰ A
NSCLC0003	F	55	N	Y	Adenocarcinoma	1.5 cm	T1	N0	M0	Ⅰ A

NSCLC0004	F	48	N	Y	Adenocarcinoma	1.1 cm	T1	N0	M0	Ⅰ A
NSCLC0005	M	68	N	Y	Adenocarcinoma	2.1 cm	T1c	N0	M0	Ⅰ A
NSCLC0006	M	64	N	Y	Adenocarcinoma	1.6 cm	T1b	N0	M0	Ⅰ A
NSCLC0007	F	78	N	Y	Adenocarcinoma	3 cm	T2	N0	M0	Ⅰ B
NSCLC0008	M	67	N	Y	Adenocarcinoma	3 cm	T2	N0	M0	Ⅰ B
NSCLC0009	F	61	N	Y	Adenocarcinoma	2.4 cm	T2	N0	M0	Ⅰ B
NSCLC0010	F	66	N	Y	Adenocarcinoma	2.8 cm	T2	N0	M0	Ⅰ B
NSCLC0011	M	62	N	Y	Adenocarcinoma	4.3 cm	T2	N0	M0	ⅡA
NSCLC0012	F	55	N	Y	Adenocarcinoma	3 cm	T2	N1	M0	ⅡB
NSCLC0013	M	70	N	Y	Adenocarcinoma	3 cm	T1	N1	M0	ⅡB
NSCLC0014	M	59	N	Y	Adenocarcinoma	4.7 cm	T2	N1	M0	ⅡB
NSCLC0015	M	61	N	Y	Adenocarcinoma	3.5 cm	T2	N1	M0	ⅡB
NSCLC0016	M	67	N	Y	Adenocarcinoma	2.4 cm	T2	N1	M0	ⅡB
NSCLC0017	F	52	N	Y	Adenocarcinoma	3.6	T2a	N1	M0	ⅡB
NSCLC0018	F	65	N	Y	Adenocarcinoma	4 cm	T2b	N1	M0	ⅡB
NSCLC0019	M	63	N	Y	Adenocarcinoma	5.5 cm	T3	N0	M0	ⅡB
NSCLC0020	F	37	N	Y	Adenocarcinoma	2	T2a	N2	M0	ⅢA
NSCLC0021	M	47	N	Y	Adenocarcinoma	2.8 cm	T2	N3	M0	ⅢB
NSCLC0022	M	54	Spine	Y	Adenocarcinoma	1.6 cm	T2	N0	M1	Ⅳ
NSCLC0023	F	78	N	Y	Adenocarcinoma	1.5 cm	T3	N0	M1a	Ⅳ
NSCLC0024	M	52	N	Y	Adenocarcinoma	0.5 cm	T3	N0	M1a	Ⅳ
NSCLC0025	F	61	N	Y	Adenocarcinoma	2.5 cm	T3	N3	M1a	Ⅳ
NSCLC0026	F	56	N	Y	Adenocarcinoma	4 cm	T3	N1	M1a	Ⅳ
NSCLC0027	F	66	N	Y	Adenocarcinoma	3 cm	T3	N2	M1a	Ⅳ
